# Barriers to effective uptake and provision of immunization in a rural district in Uganda

**DOI:** 10.1371/journal.pone.0212270

**Published:** 2019-02-14

**Authors:** Oliver Ombeva Malande, Deogratias Munube, Rachel Nakatugga Afaayo, Kisakye Annet, Bongomin Bodo, Andrew Bakainaga, Elizabeth Ayebare, Sam Njunwamukama, Edison Arwanire Mworozi, Andrew Munyalo Musyoki

**Affiliations:** 1 East Africa Centre for Vaccines and Immunization (ECAVI), Kampala, Uganda; 2 Makerere University, Department of Paediatrics & Child Health, Kampala, Uganda; 3 Egerton University, Department of Paediatrics & Child Health, Nakuru, Kenya; 4 Mulago National Referral Hospital, Directorate of Paediatrics & Child Health, Kampala, Uganda; 5 World Health Organization (WHO), Kampala, Uganda; 6 Makerere University, Department of Nursing, Kampala, Uganda; 7 Sefako Makgatho Health Sciences University, Pretoria, South Africa; University of Sydney, AUSTRALIA

## Abstract

**Introduction:**

Hoima, one of the largest districts in mid- western Uganda, has persistently performed poorly with low immunization coverage, high immunization drop outs rates and repeated outbreaks of vaccine preventable diseases especially measles. The objectives of this study were to evaluate the state of immunization services and to identify the gaps in immunization health systems that contribute to low uptake and completion of immunization schedules in Hoima District.

**Methods:**

This was a cross sectional mixed methods study, utilizing both qualitative and quantitative approaches. A situation analysis of the immunization services was carried out using in-depth interviews with vaccinators, focus group discussions and key informant interviews with ethno-videography. Secondary data was sourced from records at headquarters and vaccination centres within Hoima District. The quantitative component utilized cluster random sampling with sample size estimated using the World Health Organization’s 30 cluster sampling technique.

**Results:**

A total of 311 caretaker/child pairs were included in the study. Immunization completion among children of age at least 12 months was 95% for BCG, 96% for OPV0, 93% for DPT1, 84.5% for DPT2, 81% for DPT3 and 65.5% for measles vaccines. Access to immunization centres is difficult due to poor road terrain, which affects effectiveness of outreach program, support supervision, mentorship and timely delivery of immunization program support supplies especially refrigerator gas and vaccines. Some facilities are under-equipped to effectively support the program. Adverse Events Following Immunization (AEFI) identification, reporting and management is poorly understood.

**Conclusion:**

Immunization services in Hoima District require urgent improvement in the following areas: vaccine supply, expanding service delivery points, more health workers, transport and tailored mechanisms to ensure adequate communication between health workers and caretakers.

## Introduction

Vaccine preventable diseases (VPDs) continue to be an important public health problem in developing countries [1], making immunization a reliable child survival strategy, that prevents more than 2.5 million child deaths each year [1–4]. Approximately 10 million under five deaths occur in low-income countries annually [3]. Immunization is therefore a key intervention towards attaining Sustainable Development Goal (SDG) number 3 that aims at reduction of under-five mortality to less than 25/1000 live births by 2030 [5]. While global progress has been made to ensure provision of childhood vaccinations, difficulties still exist especially on how to reach the most vulnerable, poorest, disadvantaged childhood populations in remote communities, especially within sub-Saharan Africa [5, 6]. The reach every child (REC)/ reach every district (RED) concept was introduced to ensure that all children receive their vaccination at all levels [1]. Drivers of immunization inequities in vaccine coverage across populations include low education level of parents/caretakers, cultural/religious beliefs, age of caretakers, terrain, accessibility to health facilities, refugee status, mobility of populations, negative messaging/anti-vaccine sentiments and social economic status and attitudes of the parents/caretakers [6–11]. Studies done in Uganda have found that the level of education of the caretakers, awareness of availability of immunization services, health seeking behavior and distance to the service delivery points are the main factors contributing to low immunization coverage. The ministry of health has instituted strategies like radio talk shows, mass campaigns, static and outreach programmes in a bid to change socio cultural, religious beliefs and attitudes towards immunization but with little success. Besides, there could be variations in health information management system (HMIS) based estimates of childhood immunization coverage and actual rates at community level. In addition, the HMIS based estimates do not reflect the factors associated with low immunization coverage [3,9,12,13].

In Uganda, HMIS estimates that only 55% of children aged 12–23 months were found to be fully vaccinated with coverage being relatively higher in urban areas (61%) than rural areas (50%). Notable health challenges influencing and hindering effective immunization services in most low-income countries like Uganda include poor transport terrain, inadequate stock to cover existing demand in some facilities, frequent vaccine supply stock outs, inadequately trained health care staff, understaffing of health care workers and low knowledge of the communities about available vaccines and need for vaccination, [14–17].

While Uganda has a mandatory and active National Immunization Program, with laws that encourage immunization and that criminalize intentional failure of caretakers to take their children for routine immunization, Hoima, one of the largest districts in western Uganda bordering the Democratic Republic of Congo (DRC), has persistently performed poorly with low routine immunization coverage. The district continues to frequently report outbreaks of vaccine preventable diseases especially measles. Hoima still has challenges in achieving a DPT3 immunization coverage of >90%. For the fiscal year 2013/14, the district DPT3 and measles 9-month dose vaccination coverage was 73% and 68% respectively. This was below the national targets of 90% and 95% respectively. An understanding of the status of immunization in Hoima District, the underlying facilitators and barriers to effective immunization in the District can provide useful information to inform policy on improving immunization in Uganda, provide baseline information for designing relevant interventional studies and trials and modeling studies addressing these problems, the findings of which can then be escalated to cover the entire country and similar low-income populations especially in Sub Saharan Africa. The objectives of this study were to evaluate the state of immunization services and to identify the gaps in immunization health systems that contribute to low uptake and completion of immunization schedules in Hoima District.

## Methods

This was a cross sectional mixed methods study, utilizing both qualitative and quantitative data collection methods. This study was conducted between June and August 2017, in Hoima District in mid-western Uganda. This is a rural mixed population District, with a significant part of the population comprising of refugees and the mobile migrant border community along the Congo border. The district, largely comprising farmers and fishermen, has 13 sub counties, 54 parishes, 643 villages, served by two health sub districts with 54 Health facilities all together. A review of the state of the District immunization services was carried out using In-depth interviews (IDI) with vaccinators, community leaders and child caregivers from Hoima district. A total of 311 child-caretaker pairs were randomly selected from participants who visited health facilities for routine immunization at either the 6, 10, 14 weeks and 9 months immunization visits, and upon consenting to participate, were subjected to interviewer guided questionnaires. A separate set of 311 child-caretaker pairs were randomly selected at health facilities among mothers/caretakers whose infants possessed a well-kept vaccination book and had attained an age of 12 months and thus were expected to have completed their immunization schedule (which in Uganda ends at measles vaccine at 9 months). This set of 311 pairs of participants were assessed and analyzed only for vaccination completion rates. Data was collected by trained research assistants under the guidance of the principal investigators and collaborators. The entire data collection, facility visit and interview exercise was supervised by the principal investigators and co-authors, who were also study collaborators. Secondary data was sourced from records at District offices, and vaccination (static and outreach) data within Hoima District. Sample size was estimated using the World Health Organization’s (WHO) 30 cluster sampling technique for cluster survey design [18–19]. A multi-stage cluster sampling method was used that involved all the 13 sub-counties and 54 parishes. A parish constituted a cluster to ensure a wide distribution across the district. From each of the clusters, minimum sample of 5 caretaker-child pairs was selected by convenience (consent by caretaker to participate in the study). These were then selected from active immunization centres in the parish. Data regarding vaccination completion rates was based on immunization cards and clinic records to evaluate completion rates for all children who have made at least one year of age, and who ideally should by then have completed the recommended immunization schedule from birth to nine months in Uganda. School and facility (outreach and static) registers were reviewed, to compare uptake of HPV vaccines for girls aged 10–14 years, for the first and second dose. The health workers responsible for carrying out these immunization activities and key informants were interviewed on the general uptake for dose 1 and 2, completion rates and barriers to the roll out of HPV vaccination in the district. Focus group participants were chosen through purposive sampling in order to achieve maximum variation in the sample.

### Guide for focus group discussions

Each Focus group had around 10 participants (8–12), with a moderator and note taker, and recording of the proceedings was done. Each participant provided informed consent to participate in the focus groups and for recording to be done. The questions covered in the focus groups are as shown in Table 1.

**Table 1:** Question guide for focus group discussions.

No.	Focus group question
**1**	Why do you think children are given vaccines/immunization?
**2**	Are there situations when you failed to bring your child for immunization? What were the reasons?
**3**	Do you think that most parents from your area accept taking their children for immunization? Are there those who do not? What are some of the reasons why they opt not to take the children for immunization?
**4**	Are there days you went to a health facility and found when there were no vaccines? Which vaccine was it? And what did you do to get your child vaccinated?
**5**	Are there any side effects to vaccines? Has your child or a child you know of ever got those side effects? What did the parent or caretaker do to help the affected child?
**6**	Are there any religious groups or cultural groups you know of, (maybe not from your area) that do not encourage or promote immunization for children? If yes, what are their reasons for being against vaccines/immunization?
**7**	What is the first thing the government does when they want to introduce a new vaccine to your area? Do they educate the community enough? Do they usually get feedback from the community?
**8**	What can be done or in what ways do you think parents/mothers from your locality can be better empowered or helped to demand for or access immunization services?

### Participant consent and ethical approval

Ethical approval was obtained from the School of Medicine, Research and Ethics Committee (SOMREC), Makerere University College of Health Sciences, and the Uganda National Council of Science and Technology (UNCST), SOMREC IRB (REC REF 2017–077), UNCST (SS4245). The study involved four different aspects of consent. Separate individual written informed consent was sought for each participant/caretaker interviewed; for each participant included in each focus group, for each participant interviewed as a key informant, and by parents/caretakers of all minors. Separate written consent was obtained from the Uganda National Council for Science and Technology (UNCST) and Office of the President to allow for access to and utilization of secondary health data and immunization/population statistics in this study. Where additional information was sought that was not covered by these levels of consent, the need for consent was waived by the Makerere University School of Medicine Research and Ethics Committee (SOMREC) REF 2017–077.

### Data management

Findings from focus groups, key informant interviews and video-tapes were transcribed into Microsoft Word 2010 by the research assistants who were fluent in both the local languages and English. After transcription, team members compared each video-tape to its respective transcript and notes taken in the field by note takers for accuracy. Analysis was undertaken by two behavioural scientists experienced in qualitative research. The transcripts were then imported into the NVIVO 12 program to explore both anticipated and emergent themes, using thematic analysis techniques. For the group discussions, the facilitator and another member of the research team analysed the transcripts and met regularly to discuss the coding frame; the transition from open codes to themes and sub-themes; and the definitions and relationships between the latter. All quantitative data (including secondary data) was collected and entered anonymously in a Microsoft Excel spreadsheet and exported to Stata version 13 for analysis. Descriptive analysis of the study sample was expressed as means ± standard deviation, frequencies and percentages. Care was taken to ensure no harm or undue exposure of participants. Data and information collected during the course of the study has been safely stored.

## Results

The study successfully enrolled and analyzed 311 caretaker/child pairs in focus group discussions and another 311 participants for completeness of their immunization schedule. From the 311 caretaker/child pairs enrolled, the majority 95% (295/311) were female; either catholic or protestants (33.4% vs 28.9%); and married 87.8% (273/311). Other caretaker characteristics are as shown in Table 2.

**Table 2:** Characteristics of caretakers.

Variable	Frequency (N = 311)	Percentage (%)
**Relationship to the participant child**		
Parent	309	99.3%
Others	2	0.7%
**Age**		
< = 25	184	59.1%
26–30	63	20.2%
31–35	32	10.2%
36–40	21	6.7%
>40	11	3.6%
**Sex**		
Male	16	5.1%
Female	295	94.9%
**Religion**		
Muslim	13	4.2%
Catholic	137	44.1%
Protestant	116	37.3%
Pentecostal	39	12.5%
Traditional (Bisaka)	3	1%
SDA	3	1%
**Marital status**		
Married	273	87.8%
Single	19	6.1%
Widowed	3	1%
Divorced	16	5.1%
**Occupation**		
Housewife	13	4.2%
Peasant farmer	199	64%
Employed/professional/others	22	7%
Self-employed	69	22.2%
None	8	2.6%
**Education level**		
None	18	5.8%
Primary	191	61.4%
Secondary	86	27.7%
Tertiary	16	5.1%
**Is the caretaker household head**		
No	249	80%
Yes	62	20%
**Occupation of household head**		
Professionals	25	8%
Self employed	61	19.6%
Peasant farmer/housewife	163	52.5
None	62	20
**Sex of the household head**		
Male	270	86.8
Female	41	13.2

As regards the characteristics of children surveyed, majority 46% (145/311) were aged less than 3 months; 33.8% (105/311) were between 3–12 months, while 19.6% (151/311) were of age above 12 (13–60) months. 84.9% (264/311) /of children included in the study were born at a health facility, while 12.9% (40/311) were born at home, and the rest 2.3% (7/311) were born on the way to hospital. Regarding whether a child had been assessed for immunization status on any previous visit to a health facility, 53.7% (167/311) of respondents answered in the affirmative. Majority 99% (308/311) of respondents sought treatment from a health facility when sick (20% from hospital, 73% from dispensary/health Centre, and 19% from private clinics).

As shown in Table 3, a total of 311 child/caretaker pairs of participants were assessed and analyzed only for vaccination completion rates. Their records in the immunization card/book were compared to clinic record for correctness by two separate research assistants. The vaccine completion rates for various recommended age specific vaccines was 95% for BCG, 96% for OPV0, 93% for DPT1, 84.5% for DPT2, 81% for DPT3 and 65.5% for measles (Table 3).

**Table 3:** Completion of immunization schedule for children aged above 12 months.

Antigen	Frequency (N = 311)	Age at Vaccination	Percentage (%)
**BCG**		Birth	
Yes	296		95%
No	15		5%
**OPV0**		Birth	
Yes	299		96%
No	12		4%
**DPT1**		6 Weeks	
Yes	291		93%
No	20		7%
**DPT2**		10 Weeks	
Yes	263		84.5%
No	48		15.5%
**DPT3**		14 Weeks	
Yes	252		81%
No	59		19%
**Measles**		9 Months	
Yes	204		65.5%
No	107		34.5%

**Fig 1:**
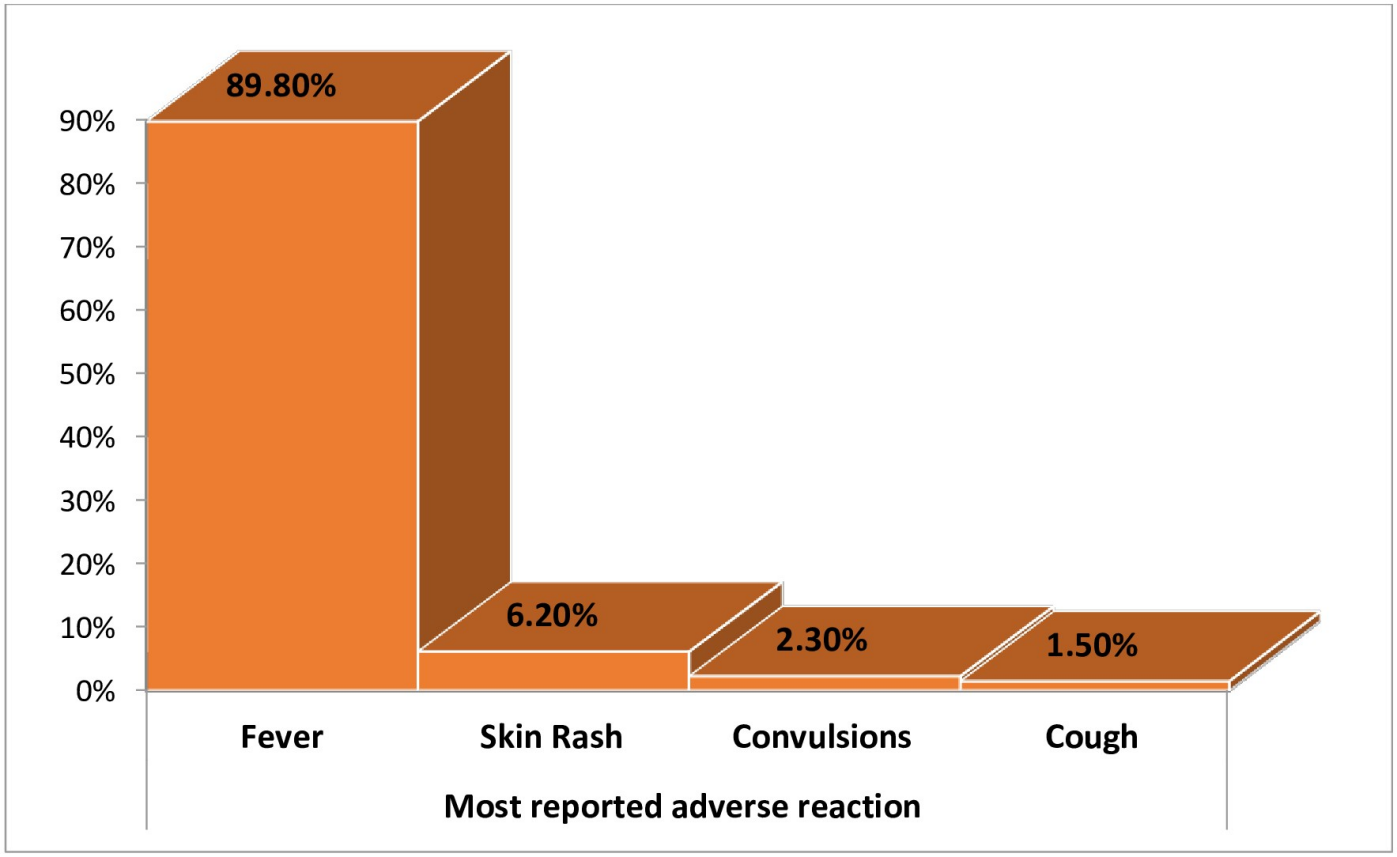
Nature of adverse event previously observed.

As shown in Fig 1, a significant proportion of respondents 41.2% (128/311) had observed a possible AEFI after immunization. It was noted that possible non-serious or self-resolving incidents of AEFIs most likely often go unreported unless probed, and this could possibly explain this fairly high percentage of AEFIs since these were based on actual probing and inquiry to the participants. These were results based on responses from the quantitative component for caretakers interviewed at immunization sites. These findings were also explored at the focus groups for caretakers and key informant interviews for health workers. Out of the 128 respondents, majority 89.8% (115/128) developed a fever after receiving the vaccination, followed by skin rash 6.2% (8/128), convulsions 2.3% (3/128), and cough 1.5% (2/128). These responses were similar to those given below from the group discussions and key informant interviews regarding AEFIs.

### Findings from the focus groups and key informant interviews

This study also carried out in-depth interviews and group discussions with mothers, community leaders, VHTs and Health workers about the status of immunization services in Hoima district, gaps in the immunization system and solutions to the challenges. A total of 13 focus groups were conducted, involving caretakers. Each focus group comprised 10–12 caretakers, a total of 40 key informant interviews were conducted. These involved community leaders (Village Health Teams, [VHTs] Local Council [LC] chair persons, secretaries, treasurers, heads of health facilities, cold chain technicians, and members of the District Health Team [DHT]) and key opinion leaders). Thirty five percent of the key informants were female, with a median working experience of 4.5 (1–10) years. The focus area for the group discussions included (but not limited to) the following questions: why do you think children are given vaccines/immunization; are there situations when you failed to bring your child for immunization—and what were the reasons; do you think that most parents from your area accept taking their children for immunization; are there those who do not—and what are some of the reasons why they opt not to take the children for immunization; are there days you went to a health facility and found when there were no vaccines—which vaccine was it—and what did you do to get your child vaccinated; are there any side effects to vaccines and has your child or a child you know of ever got those side effects—what did the parent or caretaker do to help the affected child; are there any religious groups or cultural groups you know of, (maybe not from your area) that do not encourage or promote immunization for children—if yes, what are their reasons for being against vaccines/immunization; what is the first thing the government does when they want to introduce a new vaccine to your area—do they educate the community enough—do they usually get feedback from the community—and what can be done or in what ways do you think parents/mothers from your locality can be better empowered or helped to demand for or access immunization services? The findings of the group discussions and key informant interviews are summarized below:

#### Status analysis of the immunization services

Respondents were interviewed on the overall performance of immunization in Hoima district. The number of children immunized daily was big ranging from 50 at a health center (HC) II up to 200 at the regional referral hospital (RRH). Although most health centres conduct outreach, the main regional referral hospital does not engage in outreach services.

*“We carry out immunizations on Thursday for static and Tuesday for outreach sessions*. *In a month we immunize between 150–200 babies*. *We do outreach to ease access to immunization services*, *because there some places which are far from the facility*, *so we realized the mothers used to miss out on immunization because they can’t move up to the facility because of the long distance”*.

*EPI focal person*, *at a HC III*.

*“We do immunization daily and we don’t conduct out reaches*. *We immunize between 450 and 1*,*000 children a month”*.

*EPI Key informant from RRH*.

Health workers organize their schedules to ensure coverage of immunization services at all levels of the health system. In most cases only one nurse/midwife is allocated to offer immunizations services irrespective of the number of mothers attending at a facility. This reveals a shortage of staff as the respondents below said: “*We have a shortage but we rotate so that we have at least one person in the immunization clinic*.*” An EPI focal person*, *HC II*

“*We have 2 midwives and 4 nurses*, *who cover their hospital duties including immunization*, *antenatal and deliveries*, *one is assigned to cover the immunization clinic at a time*, *and the records personnel helps us especially during the outreach”*

*A KI from the RRH*.

Respondents were asked whether caretakers have reported any Adverse Events Following Immunization (AEFIs), how often this happens, and whether there is a standardized reporting system for AEFIs. Some of the AEFIs reported included fever, injection site swelling, skin rash, abscesses, convulsions and cough. There was a lack of knowledge regarding the expected AEFIs for the different vaccines as what was mentioned was merely due to poor injection practices.

*“I saw one child who got an abscess*. *We have also received around 3 cases who got swelling at injection site when we gave pneumococcal vaccine. We have a booklet for recording these adverse effects”*

a focal person from a HC IV

*“Yes*, *we had a problem two months ago who came with fever and swelling*. *It was attributed to DPT… as for reporting*, *we have a system*, *though it’s not standardized and it’s what we are working on*. *We think the problem occurred because of the technique that could have been used*. *We discussed it in our meeting and it has never re occurred”*.

*HC III immunization focal person*.

There were misconceptions of what should happen after possible adverse events following particular vaccines. One of the mothers in a focus group said: *“The child got fever for one or two days*. *My child’s thigh got swollen after the injection*, *I came to the nurse and she told me that all will be well that instead when the child doesn’t swell; it means that the vaccine is not working but the baby got well*. *I used a cold bottle and put it on the swollen part and also gave panadol”*.

The system for keeping vaccine stocks, cold chain, transport and storage was present. Respondents were aware of the supply chain from the district stores to the health facilities. Stock outs of vaccines especially BCG, IPV, measles and oral Polio was a major hindrance to immunization service delivery. The stock outs were frequent occurrences as voiced by respondents from several facilities.

*“Vaccines are supplied from the district stores*. *The Vaccine supply is inadequate and we experience stock outs; right now*, *we don’t have BCG*. *IPV is most affected*. *The stock out can take between a week to a month”*.

*Immunization focal person*, *HC IV*

*“We get stock outs like every 2 months*. *The most affected vaccines with stock outs are Polio and IPV especially during April….”*

*EPI focal person*, *HC III*

The health facility near Lake Albert landing site had stock out problems of BCG vaccine to be given a birth as quoted below:

“*In the past few months*, *we have had a problem with BCG vaccines*. *We have also lacked measles vaccines*. *We do experience stock outs often*, *every quarter we can have a stock out twice*. *BCG is mostly out of stock”*.

*Nurse*, *HC III*

#### Barriers/gaps in immunization health systems that contribute to low uptake and completion of immunization schedules in Hoima District

The distance some caretakers have to cover to reach some immunization centres is long. This also means that health workers performing outreach services have to travel longer distances, which can be challenging when transport facilitation is inadequate and the geographical terrain is difficult to be covered. Some health facilities in geographically long sub counties are too big to be covered by the available health facilities. Generally, a number of Health workers in Hoima pointed out transport as an issue towards effective immunization. A community leader from Kabwoya gave the following explanation as one of the reasons for caretakers missing immunization appointments:

*“We lack health centres*, *the maternal health project ended*, *therefore when it comes to outreach services*, *it is difficult for the health workers to use their own earned money to transport themselves to the community for immunization*. *The people in this area are poor*, *because someone cannot afford transport to the facility; they fail to make it for immunization*. *The government passed a policy that there should be a health centre II for every parish but in Kabwoya it is not the case*, *Kabwoya health centre III serves three parishes and it’s hard for all the mothers to travel across villages to come for immunization because it involves transport costs*, *and if a mother cannot afford the transport*, *they end up not coming for immunization*. *Since this is where the oil is*, *we have a high population therefore the facilities available cannot serve the large population”*.

Language barrier is a major problem especially in the refugee camp and areas bordering Congo. People from diverse origins who speak different languages have infiltrated Hoima. Especially in areas where there are refugees and landing sites. The focal person from Rwenyawawa Health Centre III which is in the refugee camp settlement said:

*“We have a challenge of language barrier especially me who has grown up in the west I only know Runyakole*, *Rukiga and Runyakitara*, *I don’t understand the northern language*, *Congolese and Kiswahili*, *I would communicate to them using English but they don’t understand it*, *however we try to cope up and learn a few words”*.

The same barrier was also highlighted from Tonya:

“*We have a problem of language barrier as most of our patients are Alurs and they don’t speak Runyoro they only speak Kiswahili and Alur which makes our interventions hard because we don’t understand each other”*.

One of the supporting factors to the effective immunization services is the positive attitude of health workers towards the service. However, some of the community leaders and village health team members considered inadequate health worker staffing numbers, vaccine stock outs as significant gaps affecting immunization service delivery, as seen from the responses below:

*“We have a big population; the people have challenge of distance and transport and some cannot reach the outreach sites. We have a challenge in the staffing, and stock out of vaccines at the HC III, where for the last two weeks they do not have some vaccines”*.

From A Community leader. Similar sentiments were captured from Rwenyawawa Health Centre III in the refugee camp settlement.

*“Sometimes we go and they tell us the drugs are not available*, *that we should come back another time*, *the drugs for babies especially BCG*. *Even the drugs are not there*. *“Even me mine of 9 months has never received it” You can walk a very long distance yet when you reach here*, *they send you back*. *“Even me mine is 2 months and has never received it*. *In fact*, *they couldn’t give my child today*.*” When they are not there*, *I walked to Kafuba*, *the neighbor health center”*.

A caretaker at a focus group discussion at Kigorobya HC IV.

In relation to religion and cultural influence as barriers to effective immunization services, one of the community leaders said:

*“We had a religion that believe that people should not take their children for immunization but we have fought that mentality out of the people*. *They give reasons that Jesus was not immunized and there is no evidence that Jesus was immunized and that the vaccines are harmful”*.

“*Yes*, *there is one religious which is a cult “egiri ya Yesu”*, *they don’t immunize their children*. *Their leaders just tell them not to take children for immunization or treatment”*.

A caretaker during a focus group discussion at Buhanika.

*“Their God (owobusobozi) stops them from being the children for immunization”*.

A caretaker during a focus group discussion at Kikube HC III.

*“The religion of Bisaaka people do not believe in immunization*. *There is a new religion that has been around for like a week*, *that does not encourage people to immunize children*. *They say that the people who were not immunized have lived longer and the people are immunized die earlier”*.

A community leader during a focus group discussion.

Some respondents reported inadequate male partner involvement as a barrier to effective immunization. A caretaker during a focus group discussion said:

*“Some men tell the wives that “where are you taking my child? The child over cries the entire night and makes noise for me”*.

## Discussion

In this cross-sectional study, we found that the major barriers to effective immunization in Hoima District were vaccine stock outs, access (transport difficulties/ long distance to health facilities/a difficult geographical terrain) and language barrier especially affecting communities living along the Congo border. The hilly and mountainous rocky terrain with narrow marram roads that make it difficult to navigate and inadequate transport affect the steady supply of immunization consumables from the district stores to the various health facilities. There are a few facilities which also utilized refrigerators from neighboring health centres due to poor working condition of their vaccine fridges. Some of these facilities with fridges in poor working condition include Buraru, Kisiha, and Nsozi HCs. Hard to reach facilities especially along Congo border thus suffer increased waiting time or missed vaccination appointments as caretakers wait for vaccines to be brought. There were reports of situations where there was no readily available transport, thus the vaccines would not be collected in time for the vaccination day. The geographical terrain in most parts of Hoima district is poor, and in some areas it’s possible to go to the facility but hard to come back; a factor that also affects caretakers/parents in such regions to take their children to the health facility for immunization. They would rather stay home, additionally because the journey to and from the facility, plus waiting time will mean a whole day (6–10 hours) yet they have their farms and other competing needs to attend to. This would indirectly contribute to high dropout rates or delay in the schedule for the affected children because these caretakers may not be able to return on the next rescheduled date. As has been shown in other Ugandan studies [20–21], missed opportunities and long waiting time at static and outreach immunization centers greatly contributes to failure to complete immunization schedule. Some possible explanations advanced by these studies include having inadequate amount of vaccines supplied, or inadequate number of facilities or health workers to run the immunization services. Vaccine stock outs were a major barrier in some parts of Hoima district, as revealed by the focus groups and key informant interviews. This is similar to a study done in Yumbe, the northern part of Uganda [21–22] and the Uganda MOH Annual Health Sector Report (AHSR) 2012/13; which reported that vaccine stock outs and supply shortage problem are reasons why some caretakers fail to complete recommended immunization schedule and some districts continued to perform poorly in immunization completeness. The study found that there is demand for vaccination services except for when religious or traditional believes were sited.

While this study was not designed and specifically powered to address the question of immunization coverage in Hoima District, the performance indicators showed worrying results. The results in this group of children revealed completion rates for various vaccines at 95% for BCG, 96% for OPV0, 93% for DPT1, 84.5% for DPT2, 81% for DPT3 and 65.5% for Measles. These figures are similar to National figures for Uganda, where only 55% of children aged 12–23 months were found to be fully vaccinated with full vaccination coverage being relatively higher in urban areas (61%) than rural areas (50%) [14–16]. For the fiscal year 2013/14, the district DPT3 and measles vaccination coverage were 73% and 68% respectively, which is way below the national targets of 90% and 95%. The measles uptake of 65.5% found in this study does explain in part why recurrent measles outbreaks remain a major problem in Hoima district and could be a result of vaccine stock outs, long distance to health facilities, health workers overwhelmed at immunization clinics, lack of transport and language barrier that could affect communication between the health workers and caretakers about the need of returning for subsequent immunization visits.

Most residents of Hoima district are very receptive to vaccines and immunization services. The health workers in Hoima district are willing and committed to immunization programs. This was very positive and encouraging finding, and a departure from findings of other studies [12, 20–24] that suggested that health worker attitude was a major hinderance to caretaker uptake of immunization services. Those studies had found that health workers treated care takers in a rude way or reported late for work making the caretakers to wait for long hours at health facilities. In Nigeria, health worker negative effect extended to poor communication of vaccine health information both in content and manner in which the information was delivered, that was found not to be conducive for caretaker learning [12, 20–24].

While the district has a committed cold chain team, they face transport difficulties to effectively ensure cold chain preventative maintenance, support supervision, program mentorship and timely delivery of immunization supplies. The supply of gas to Hoima district central stores by National Medical Stores (NMS) is steady and reliable; however, delivery of the gas to the various facilities within the district is hampered by transport and funding difficulties, especially delivery to the hard to reach areas for example facilities at the lake shores. There may be a need to strengthen the “Last mile” concept where supplies are taken to the final end user/ health facility.

Most of the facilities have an outreach program plan in place; however due to lack of enough funding for transport and poor geographical and road terrain system in the district, the outreach program especially in hard to reach areas remains a challenge. For example, the outreach service in Rwenyawawa or Buseruka can have a turn up of as many as 400 children; this is overwhelming for the two-immunization staff available from the supporting Health Centre. There is as a result, long waiting time, inadequate immunization health promotion and education given to mothers/ caretakers who visit health facilities for routine immunization. The outreach venues/sites are often far places where the available/allocated transport means/funds are inadequate. In such situations, often the Primary Health Care (PHC) funds available usually caters for just one trip (to the venue and not back to the Health Facility), and the health workers involved return late in the night or the following day depending on weather.

Involving the community in their own vaccination to inspire good health seeking behavior has been documented as an important aspect for the success of the immunization programme; therefore, exploring the behavioural aspects influencing utilization of immunization services in Hoima District is equally important [12,20, 23, 24].

The existing program or system in place for identification, reporting and management of Adverse Events Following Immunization (AEFIs) is not well known by the health workers and community. The Ministry of health does have a system in plan, and there has been a delay in national roll out and implementation of this system. It is difficult to quantify the impact of small cults and religious groups previously thought to be promoting anti vaccine sentiments/ hesitancy in the district. The impact of these groups may have been lessened over time by the competing and sustained active immunization promotion programs within the district. Though the HPV vaccine has been rolled out and is available in most places, completion of the second dose is poor despite the district running both facility and school based (outreach) programs. This study is limited to the extent that these aspects of HPV vaccination program were not adequately explored. The main reasons for this poor uptake of the second dose therefore require further exploration.

## Conclusion and recommendations

Immunization services in Hoima District require urgent improvement in the areas of: vaccine supply, expanding service delivery points through outreach services, recruitment of more health workers, transportation and implementation of tailored mechanisms to ensure adequate communication between health workers and caretakers.

There is need for future studies which can test specific interventions in Hoima, which if successful, can be escalated to cover more districts and entire country with similar immunization inequities. These interventions could take the form of: 1. Training health workers with refresher/ new courses on basics of immunization / vaccines; 2. Community sensitization about importance of immunization; 3. Promotion of joint planning for immunization services using the reach every child approach between health workers and communities to be served; 4. Designing or implementing a system for identifying, reporting and management of AEFIs; 5. Designing and piloting a program that equips health workers and Village Health Teams (VHTs) to offer continuous education for care takers that runs along the static and outreach /mobile immunization sessions in the district. This can be done by providing enough information, education and communication (IEC) materials in the local languages taking note that Hoima district is one with various ethnic groups. There is need for more funding for the cold chain team members in the district, especially with reliable/steady transport means, to ensure sustained support supervision, mentorship, on-site training, reporting and supply of consumables, especially vaccines and gas to health facilities.
